# Draft Genome Sequence of the Aquatic Phosphorus-Solubilizing and -Mineralizing Bacterium *Bacillus* sp. Strain CPSM8

**DOI:** 10.1128/genomeA.01265-13

**Published:** 2014-01-30

**Authors:** Nilanjan Maitra, William B. Whitman, Saravanaraj Ayyampalayam, Srikanta Samanta, Keka Sarkar, Chinmay Bandopadhyay, M. Aftabuddin, Anil P. Sharma, Sanjib K. Manna

**Affiliations:** a Central Inland Fisheries Research Institute (I.C.A.R.), Barrackpore, Kolkata, West Bengal, India; b Department of Microbiology, University of Georgia, Athens, Georgia, USA; c Institute of Bioinformatics, University of Georgia, Athens, Georgia, USA; d Department of Microbiology, University of Kalyani, Kalyani, West Bengal, India

## Abstract

*Bacillus* sp. strain CPSM8 is an efficient solubilizer and mineralizer of phosphorus. Here, we present the 4.39-Mb draft genome sequence of the strain, providing insight into the phosphorus-releasing genes related to productivity in aquatic habitats.

## GENOME ANNOUNCEMENT

The essential nutrient phosphorus often remains at low concentrations in most freshwater environments, limiting aquatic productivity (1). Phosphorus-solubilizing microorganisms (PSM) provide one strategy for enhancing phosphorus availability in an ecologically benign manner. A number of microbial biofertilizers have been applied in agricultural soil to enhance phosphorus availability, and many of them promote plant growth (2, 3) by decreasing the harmful effects of pathogenic microbes, synthesis of phytohormones (4), N_2_ fixation, solubilization of mineral-bound phosphorus, mineralization of organic bound phosphorus, and making phosphorus available to plants (5). Of these bacteria, members of the genus *Bacillus* are important in the solubilization and mineralization of phosphorus in agricultural and aquatic environments (6, 7).

*Bacillus* sp. strain CPSM8 was isolated from sediment of the River Churni in West Bengal, India, using the National Botanical Research Institute’s phosphate growth medium (NBRIP) (8) at 30°C. This strain has moderate Ca_3_(PO_4_)_2_ solubilization activity (62.88 mg PO_4_^3-^ liter^-1^ in NBRIP medium, 7-day incubation), phytate mineralization activity (10.48 mg PO_4_^3-^ liter^-1^ in liquid phytate screening medium [9], 7-day incubation) and the potential for biofertilization application in freshwater ecosystems.

Here, we present a draft genome sequence of strain CPSM8 obtained from the Illumina HiSeq 2000 system. A total of 7,624,534 filtered reads for CPSM8 were assembled into 43 contigs (N_50_ length, 297,357 bp), with an average coverage of 125.0×, using the A5 Pipeline v (10). The genome annotations were performed by the NCBI Prokaryotic Genomes Annotation Pipeline utilizing GeneMark S (11).

The genome consists of 4,395,870 bases with 37 contigs >500 bp each and a G+C content of 45.9%. A total of 4,672 open reading frames (ORFs) were generated, of which 12 rRNA genes (5S, 23S, and 16S), 91 tRNAs genes, and a total of 4,521 protein-coding sequences (CDSs) were identified. A total of 483 subsystems were determined using the RAST server (12). The 16S rRNA gene sequence of CPSM8 is closely related to that of *Bacillus licheniformis* (99%), but other conserved genes (RNA polymerase beta subunit [*rpoB*], gyrase beta subunit [*gyrB*], and recombinase A [*recA*]) show various degrees of similarity (96 to 99%). The sequence contains genes, such as those encoding glucose dehydrogenase, citrate synthase, and lactate dehydrogenase, for the production of organic acids involved in inorganic phosphorus solubilization (13), as well as a gene for 3-phytase productions involved in phytate mineralization. The genome also contains two alkaline phosphatase and three 5′-nucleotidase genes, which may mineralize organic phosphorus compounds, four hemolysin genes, a feature of bacteria that interact with plants (14), and six genes for the synthesis and transport of bacillibactin, a high-affinity siderophore inhibiting the growth of fungal pathogens (15), contributing to the biofertilization potential of the strain. The genome contains genes for membrane transport systems, including 26 ATP-binding cassette (ABC) transporters and 12 genes for the phosphotransferase systems involved in the transport of organic acids (for phosphorus solubilization) and phosphorus across cell walls.

### Nucleotide sequence accession numbers.

The whole-genome shotgun project has been deposited at DDBJ/EMBL/GenBank under the accession no. ANNR00000000. The version described in this paper is ANNR00000000.2.
